# Supplementary figures and images for: Improved 3D cellular morphometry of Caenorhabditis elegans embryos using a refractive index matching medium
Source: PLoS One. 2020 Sep 30;15(9):e0238955. doi: 10.1371/journal.pone.0238955 (PMC7526913; doi:10.1371/journal.pone.0238955)

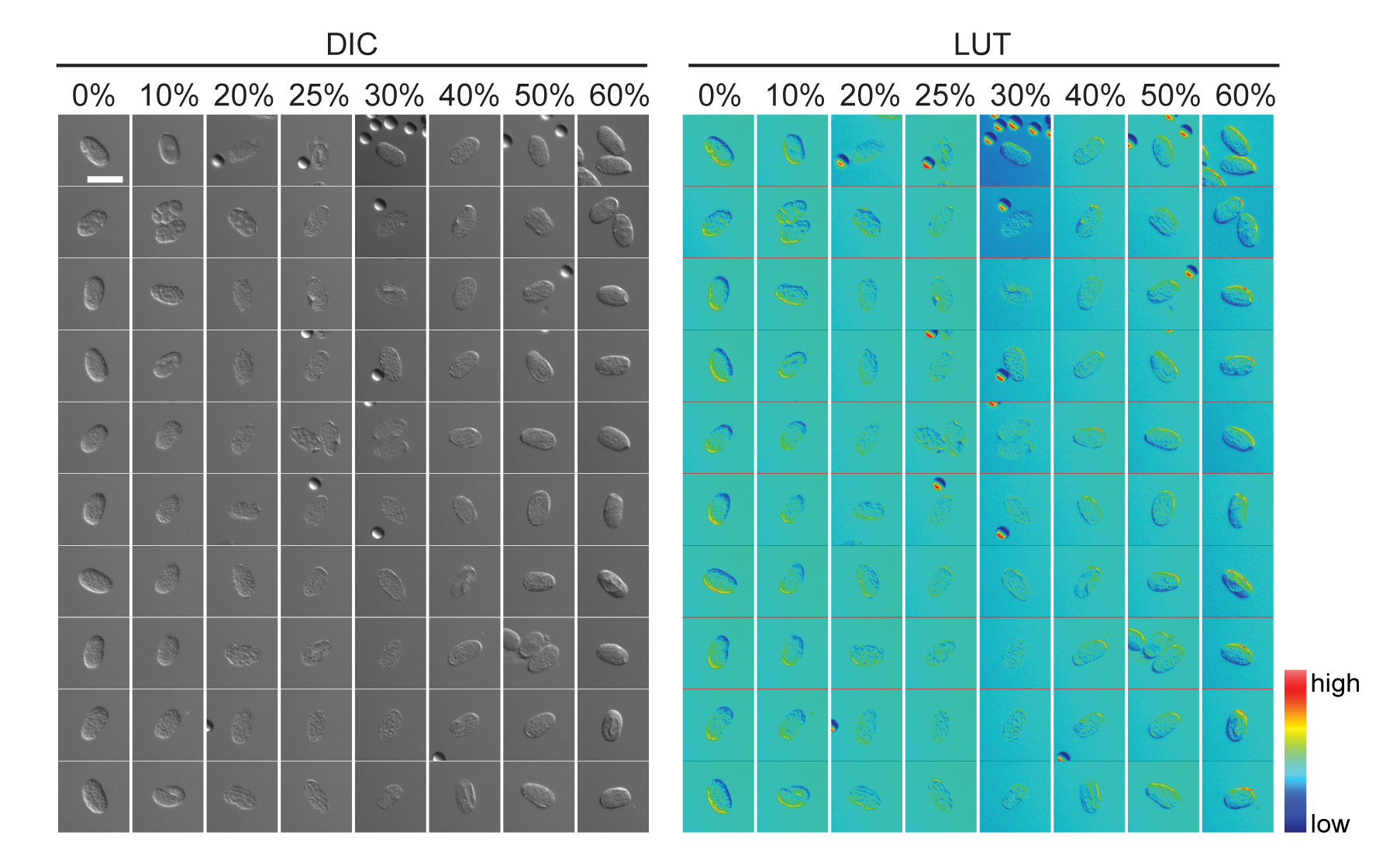

Supplement: S1 Fig — (TIF) [file pone.0238955.s001.tif]

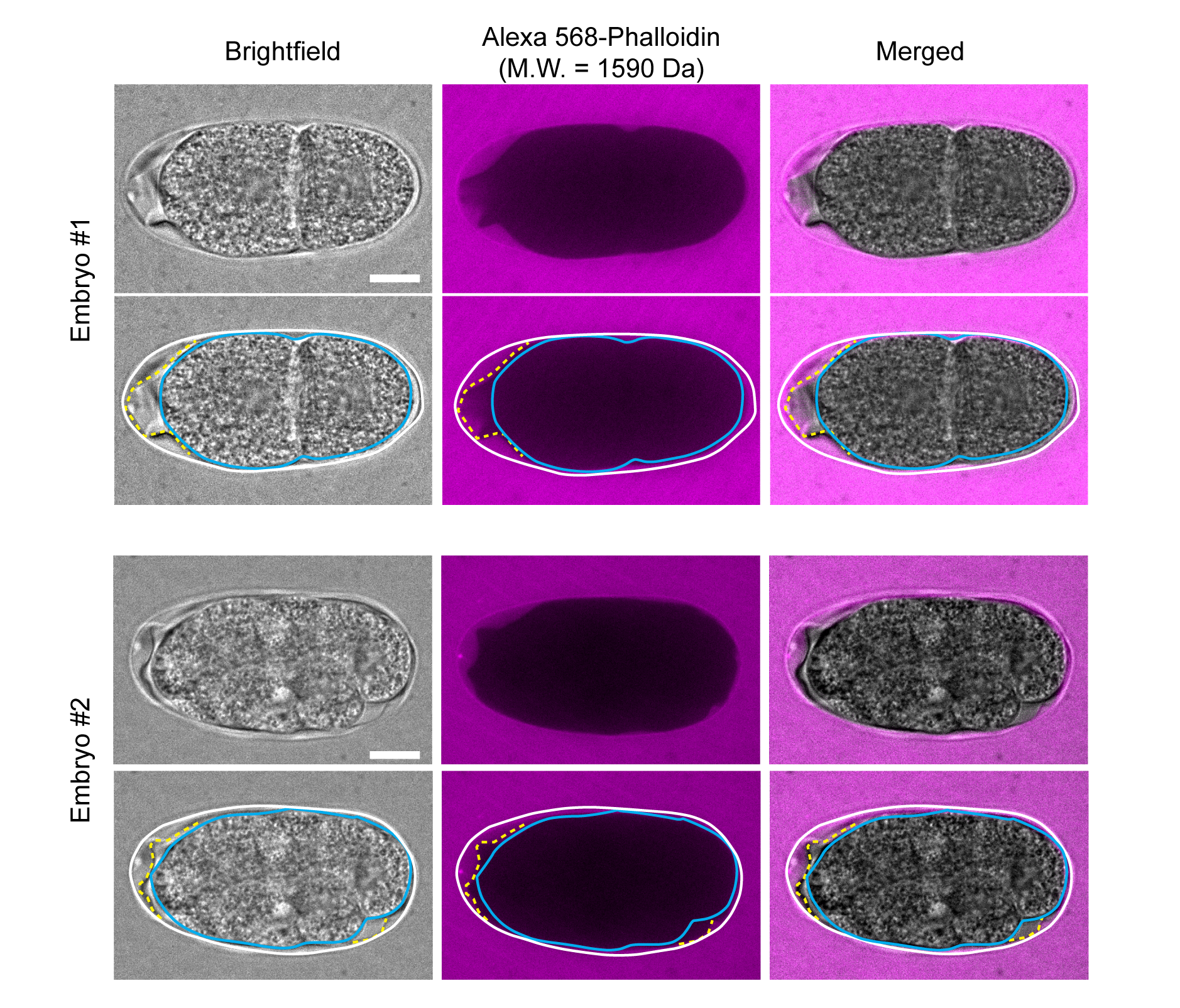

Supplement: S2 Fig — (TIF) [file pone.0238955.s002.tif]
